# Brain drain in South Africa is affecting health care

**DOI:** 10.4102/safp.v66i1.5830

**Published:** 2024-01-03

**Authors:** Indiran Govender

**Affiliations:** 1 Department Family Medicine and Primary Health Care, School of Medicine, Sefako Makgatho Health Sciences University, Pretoria, South Africa

The effective functioning of any health sector requires the availability of skilled and competent medical professionals, and South Africa is experiencing a shortage of medical professionals exacerbated by the phenomenon of ‘brain drain’, namely the depletion or loss of intellectual and technical personnel who migrate to other areas.^1^ The United Nations describes it as a one-way movement of highly skilled people that only benefits the host. Today, brain drain is a major problem facing less developed countries. Brain drain is reported to have direct negative impacts on the population’s health status in the donor country, with associated consequences for the productivity and welfare of the population.^1,2^ Many African countries face huge losses of human skills, and this, in turn, has affected their development. From health professionals to teachers, academics and engineers, the continent has lost numerous skilled personnel who ought to be contributing to its socio-economic development.^2^

A global shortage of health workers makes it relatively easy for doctors from poor countries to emigrate to rich countries. This has raised fears of a medical brain drain and has been the subject of much-impassioned debate.

Health professionals from South Africa are internationally mobile, owing to their skills acquired through long years of sound training. South Africa is argued to play a central role of ‘victim’ as well as ‘executer’ in this process. Victim because the country ‘exports’ doctors to richer countries and executer because it, in return, ‘imports’ doctors from poorer countries. Currently, many developed countries only require the Fellowship of the College of Family Physicians of South Africa (FCFP [SA]) degree from South Africa as proof of training and registration to recognise this as an equivalent of family medicine specialist training in their country. Thus, doctors may use the FCFP (SA) degree as a stepping stone, perpetuating the brain drain in Africa. The South African government frequently points to health worker migration as an exacerbating factor for the country’s health crisis. A report by the Organisation for Economic Co-operation and Development stated that there were more than 23 400 health professionals from South Africa residing and working in the United Kingdom, New Zealand, the United States and Australia.^2,3,4^ South Africa’s health sector is being crippled because it is haemorrhaging medical professionals at an alarming rate, and this is affecting the quality of healthcare provided.

Currently, about 11.13% of South Africans with higher education indicate that they are seriously considering emigrating to another country. Migration is not a simple process; it is underpinned by many push and pull factors that the potential migrant may not be able to control. Push factors are those factors that make the individual want to leave his or her place of origin; these may range from poor law enforcement, inadequate educational facilities, lack of good job opportunities, crime, political corruption and political instability among others. Pull factors are those factors that attract a person to a particular region; these include better salaries, better educational facilities, better working conditions and a stable political climate. Although the prospect of migrating to a more developed environment might be enticing for a person, there are intervening factors that may influence the decision to migrate; these include distance, costs associated with migration and family challenges.^3,4,5^ The push factors must be addressed, and these are not only financial but includes economy, living conditions, crime, prospects for a better future and government policies.^4,5,6^

South Africa has been a victim of rural–urban migration, where health workers prefer working in urban areas. Patients in rural areas walk long distances to seek medical services because doctors migrate from rural areas to urban areas, which are characterised by better working conditions and the availability of resources; this creates social injustices, and in essence, residents are also denied accessibility of quality healthcare because of the rural–urban migration problem in South Africa. It is unfortunate that the government has not dealt decisively with factors influencing the migration of skilled professionals from rural areas.^4,5,6^

Health facilities in South Africa are characterised by poor working conditions, and the salaries are sub-par. The public health facilities face the problem of migration of skilled doctors to the private sector.^2,4,5,6^ The public health sector has numerous vacancies; some have gone for years unfilled because of the unavailability of skilled medical professionals and where there are foreign nationals that qualify for such position, they may not likely be given the job because they are seen as outsiders.

Brain drain and the loss of skilled professionals are a major reason for the unsteady growth of the South African economy. The government lacks a clear-cut policy on how to reduce brain drain, and this will further impact the country’s socio-economic development. Investor confidence in South Africa is currently at all-time low, concurrent downgrades by rating agencies, corruption and poor rates of economic growth have crippled the business confidence in South Africa. Political uncertainty in South Africa is also weighing on the country’s economic direction.^2,3,5,6^

South Africa needs urgent interventions to ensure that brain drain is contained. Although it is virtually impossible to eliminate brain drain from South Africa completely, there are potential mechanisms that combine policy and monetary approaches.^4,7^ The government should increase accountability in public health institutions, as facilities are characterised by mismanagement and corruption, which undermine the efficient functioning of the health sector in South Africa.^7,8^ The government needs to improve doctors’ working conditions, security, upgrade infrastructure, ensure availability of resources as well as develop a more open immigration policy prioritising skilled immigration. To ensure inclusive access to healthcare, the government must develop an incentive mechanism that would encourage workers to stay in the rural areas or perhaps move from urban to rural areas. The government needs to increase the training of doctors; this will ensure that there are sufficient medical professionals in the system to cover any shortfall that might arise because of migration.^4,5,6,7^

The government’s national development plan that envisions South Africa as an infrastructural hub characterised by modern infrastructure that can support industrial development may remain a pipe dream if the country fails to retain its highly skilled and experienced professionals.^2,4,7,9^ Failure to do so will also jeopardise the plans for universal health coverage.
